# The Prehistoric Indian Ayurvedic Rice *Shashtika* Is an Extant Early Domesticate With a Distinct Selection History

**DOI:** 10.3389/fpls.2018.01203

**Published:** 2018-08-14

**Authors:** Mariet Jose, R. Dinesh Raj, M. R. Vinitha, Remya Madhu, George Varghese, Jan Bocianowski, Rashmi Yadav, B. C. Patra, O. N. Singh, J. C. Rana, S. Leena Kurmari, George Thomas

**Affiliations:** ^1^Plant Molecular Biology Laboratory, Rajiv Gandhi Centre for Biotechnology, Thiruvananthapuram, India; ^2^Department of Mathematical and Statistical Methods, Poznań University of Life Sciences, Poznań, Poland; ^3^ICAR-National Bureau of Plant Genetic Resources, PUSA Campus, New Delhi, India; ^4^Division of Crop Improvement, ICAR-National Rice Research Institute, Cuttack, India; ^5^National Coordinator, Bioversity International, PUSA Campus, New Delhi, India; ^6^Department of Plant Breeding and Genetics, Kerala Agricultural University, Thrissur, India

**Keywords:** Njavara, *Shashtika*, medicinal rice, ayurveda, evolution, microsatellites, sequence tagged site markers

## Abstract

Fully domesticated rice is considered to have emerged in India at approximately 2000 B.C., although its origin in India remains a contentious issue. The fast-growing 60-days rice strain described in the *Vedic* literature (1900–500 B.C.) and termed *Shashtika* (Sanskrit) or Njavara (Dravidian etymology) in Ayurveda texts including the seminal texts *Charaka Samhita* and *Sushruta Samhita* (circa 660–1000 B.C.) is a reliable extant strain among the numerous strains described in the Ayurveda literature. We here report the results of the phylogenetic analysis of Njavara accessions in relation to the cultivars belonging to the known ancestral sub-groups *indica, japonica, aromatic*, and *aus* in rice gene pool and the populations of the progenitor species *Oryza rufipogon* using genetic and gene genealogical methods. Based on neutral microsatellite markers, Njavara produced a major clade, which comprised of minor clades corresponding to the genotypic classes reported in Njavara germplasm, and was distinct from that were produced by the ancestral sub-groups. Further we performed a phylogenetic analysis using the combined sequence of 19 unlinked EST-based sequence tagged site (STS) loci with proven potential in inferring rice phylogeny. In the phylogenetic tree also the Njavara genotypic classes were clearly separated from the ancestral sub-groups. For most loci the genealogical analysis produced a high frequency central haplotype shared among most of the rice samples analyzed in the study including Njavara and a set of *O. rufipogon* accessions. The haplotypes sharing pattern with the progenitor *O*. *rufipogon* suggests a Central India–Southeast Asia origin for Njavara. Results signify that Njavara is genetically distinct in relation to the known ancestral sub-groups in rice. Further, from the phylogenetic features together with the reported morphological characteristics, it is likely that Njavara is an extant early domesticate in Indian rice gene pool, preserved in pure form over millennia by the traditional prudence in on-farm selection using 60-days maturity, because of its medicinal applications.

## Introduction

Among the numerous rice (*Oryza sativa* L.) strains described in the seminal Sanskrit compendia of Ayurveda medicine *Charaka Samhita* (Van Loon, 2003) and *Sushruta Samhita* (Bhishagratna, 1907) (circa 660–1000 B.C.) (Ninivaggi, 2010), *Shashtika* (or *Sastika*), which is grown in Kerala and known locally by the Dravidian name Njavara (Burrow and Emeneau, 1961; Singh, 1992; Sreejayan et al., 2011), is a reliable extant strain in the present day rice gene pool. The *Yajur Veda* and *Atharva Veda* (circa 1900–500 B.C.) (Ninivaggi, 2010) were the first Sanskrit texts to describe a fast-growing rice that matures at 60 days, although the authors did not use the term *Shashtika* (Nene, 2005). While, the people of Kerala have ritualistically preserved Njavara over centuries, for its grains are indispensable in the preparation of several acclaimed Ayurveda medicines (Singh, 1992; Sreejayan et al., 2011), the other rice strains described in Ayurveda texts are either extinct or we are unable to recognize them in the present day gene pool (Singh, 1992). Njavara grains are used in several Ayurveda treatments since time immemorial (Singh, 1992; Sreejayan et al., 2011). The hall mark of Njavara is its short crop duration and it matures in 60 days (Bhishagratna, 1907; Van Loon, 2003; Sreejayan et al., 2011). Farmers traditionally identify and select Njavara on-farm based on its 60-days maturity (Sreejayan et al., 2011).

Even after decades of research, the debate continues about when, where, and how many times rice was domesticated in the past. Proponents of both single origin (Molina et al., 2011; Huang X. et al., 2012) and the multiple origin (Londo et al., 2006; Civáň et al., 2016; Wang et al., 2018) models for rice domestication agree that the Indo-Gangetic plains are a major center for rice domestication. But they differ in their opinion with respect to the way by which rice was evolved in this region. As per the single origin model, fully domesticated rice was evolved in the Gangetic plains from the pre-domesticated or wild rice populations that existed here following the introgression of alleles from the fully domesticated *japonica*, which was dispersed towards the South Asia from Yangtze River basin in china (Molina et al., 2011; He et al., 2011; Huang X. et al., 2012; Yang et al., 2012). According to the multiple origin models, the fully domesticated rice was evolved in the Gangetic plains by the primary domestication from the local populations of the progenitor species *O. rufipogon* (Londo et al., 2006; Civáň et al., 2016). The archeobotanical data support the occurrence of rice in the Gangetic plains as early as circa 6000 B.C. (Saxena et al., 2006; Fuller et al., 2010; Gross and Zhao, 2014). However, it is not certain whether the *Oryza* bulliform phytoliths obtained from archeological sites in the Gangetic plains are from the cultigens or the wild rice populations (Fuller et al., 2010; Gross and Zhao, 2014). Bulliform phytolith morphometrics indicate that the development of fully domesticated rice occurred in northern India only in circa 2000 B.C. following a contact-induced hybridization between ancestral *indica* and fully domesticated *japonica* (Fuller et al., 2010). Recent studies have increasingly shown that genetic structure of rice is more complex than thought before and emphasize the importance of local gene pool in fine tuning the evolutionary trajectories of rice (Travis et al., 2015; Kim et al., 2016; Roy et al., 2016; Wang et al., 2018).

The Ayurveda texts *Charaka Samhita* and *Sushruta Samhita* represent a compilation of oral tradition of Ayurveda knowledge existed in the ancient northern India (Ninivaggi, 2010). Presumably, medicinal applications of Njavara grains must be well known when these texts as well as *Yajur Veda* and *Atharva Veda* were redacted. The supposed time period of the origin of these Sanskrit literatures (circa 1900–500 B.C.) (Ninivaggi, 2010) indicates a possible overlap with the suggested time period of the emergence of the fully domesticated rice in India (circa 2000 B.C.) (Fuller et al., 2010). Although, the archeology-genetic studies have added new insight into the domestication profile of rice (Gross and Zhao, 2014), only little effort has been so far made to employ linguistic-genetic approach to address rice domestication issues. The Njavara germplasm has not been so far subjected to a phylogenetics analysis to infer its oldness as depicted in the pre-historic Sanskrit literatures. Such phylogenetics treatment on Njavara as well as other rice strains may also provide us vital insight into the domestication profile of rice in India.

Previously, we identified six distinct genotypes in Njavara germplasm under four morphotypes (Sreejayan et al., 2011). However, the genetic relationships of Njavara to the canonical sub-groups *indica, aus, aromatic*, and *japonica* (*tropical* and *temperate*) identified in global rice gene pool (Garris et al., 2005) are not yet known, although previously we reported that Njavara is morphologically and genetically distinct from syntopic traditional cultivars grown in Kerala (Varghese et al., 2014). Another important question about Njavara is its possible center of origin. Because, although extensively mentioned in *Charaka Samhita* (Van Loon, 2003) and *Sushruta Samhita* (Bhishagratna, 1907) compiled in northern India (Ninivaggi, 2010), Njavara is currently grown and utilized for Ayurveda treatments only in Kerala State, which is located at the southern most region of India.

In this study, we asked three questions: (1) What is the position of Njavara in rice gene pool, particularly with respect to the canonical sub-groups identified in global rice gene pool (Garris et al., 2005)? (2) Do phylogenetics analyses provide a clue regarding the oldness of Njavara as depicted in prehistoric Sanskrit literatures? and (3) Where is the possible center of origin of Njavara. We analyzed 213 Njavara individuals, and 376 rice cultivars and 47 accessions of *O. rufipogon* sampled from different rice growing Southeast Asian countries using microsatellite markers, gene genealogy, and chloroplast genotyping. The data were rigorously analyzed and examined the genetic and phylogenetic interrelationships between Njavara and other sample sets.

## Materials and Methods

### Plant Materials

**FIGURE 1:**
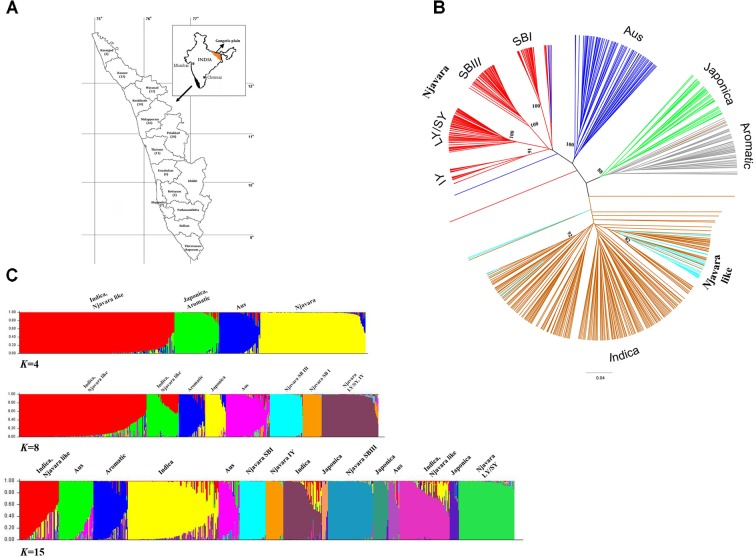
Njavara represents a distinct clade in relation to the known ancestral sub-groups in the rice gene pool. **(A)** Map of Kerala State showing different districts, with the number of Njavara samples used in the study from each district in parenthesis. **(B)** Dendrogram showing the genetic relationship between the 589 rice samples based on the allelic data generated by 70 microsatellite markers. The clusters were identified by using IRRI reference lines, and they are color coded. The genotypic classes identified in the Njavara gene pool *viz*., Intermediate yellow (IY), Long yellow/Short yellow (LY/SY), Short black I (SB I), and Short black III (SB III) are shown. Njavara-like individuals, which represent individuals from Njavara collections that clustered with ancestral groups, are also color coded. Bootstrap values > 50% are given at the respective nodes. **(C)** Bar plot showing model-based population assignment of 589 rice samples revealed by the Bayesian algorithm STRUCTURE based on microsatellite data at *K* = 4, *K* = 8, and *K* = 15. The name of the population sub-divisions identified in the analysis is shown above the bar plot produced at the respective *K-*values.

In this study, we analyzed 636 samples in total, and they included 589 accessions of Asian cultivated rice *O. sativa* L. and 47 accessions of the progenitor *O. rufipogon* Griff. The sample details are as follows: (1) *O. sativa cv.* Njavara: We began this study with 100 collections of Njavara, including the 80 collections reported earlier (Sreejayan et al., 2011; Varghese et al., 2014). The geographic origin of the collections is shown in **Figure 1A**. Sreejayan et al. (2011) assessed the intra-collection genetic heterogeneity in 28 Njavara collections and identified 42 genetically distinct individuals. In this study, we first performed the intra-collection heterogeneity assessment of the remaining 72 collections as described in the **Supplementary Table 1a** and then identified 171 genetically distinct individuals from 70 collections. Two collections were duplicates and thus were discarded. Thus, the Njavara germplasm used in this study for genetic analysis consisted of 213 genetically distinct individuals including the 42 previously identified individuals (Sreejayan et al., 2011) and the 171 individuals identified in this study (**Supplementary Tables 1a, 2**). (2) Traditional rice cultivars: We sampled 310 traditional rice cultivars from 14 states encompassing the major rice growing regions in India (**Supplementary Table 3a**). (3) Improved rice varieties: 22 improved rice varieties commonly cultivated in Kerala State were included in the analysis to test whether seed mixing or genetic admixing occurred between these common varieties and Njavara (**Supplementary Table 3b**). (4) Reference lines: Together, the isozyme (Glaszmann, 1987) and microsatellite analyses (Garris et al., 2005) classified the global rice gene pool into five ancestral sub-groups: *indica, aus, aromatic*, and *japonica* (*tropical* and *temperate*). We obtained 44 well-classified rice cultivars belonging to the different sub-groups (Glaszmann, 1987; Garris et al., 2005) from the International Rice Research Institute (IRRI), Manila, Philippines (**Supplementary Table 3c**) and used them as reference lines to identify the clusters and clades produced in different genetic analyses. The reference cultivars are originated from 17 countries, including 15 predominantly rice growing countries in South or Southeast Asia (**Supplementary Table 3c**). (**5**) *O. rufipogon*: We used 47 *O. rufipogon* accessions originating from 11 South or Southeast Asian countries and 10 states in India. Of the 47 accessions, 41 accessions were obtained from IRRI, and the remaining six accessions were sampled from Kerala State by the investigating group (**Supplementary Table 3d**).

### Microsatellite Analysis

Genomic DNA was extracted from a single plant using a GenElute Plant Genomic DNA Miniprep Kit (Sigma Genosys, Bangaluru, India) following the manufacturer’s instructions.

We selected 340 microsatellite markers covering all the 12 chromosomes of rice (**Supplementary Table 1b**) from the public database Gramene^1^, and the oligonucleotide primers were custom synthesized by Sigma (Sigma Genosys, Bangaluru, India). We performed an initial evaluation of these loci as described in the **Supplementary Table 1b**. We selected 70 loci that yielded discrete, unambiguous, and polymorphic alleles (**Supplementary Table 4a**) and genotyped 589 rice samples (**Supplementary Tables 2, 3a–c**). The loci were amplified according to the methods of Schuelke (2000). In each PCR assay, this method uses three primers: a sequence specific forward primer with universal M13 sequence (TGTAAAACGACGGCCAGT) attached to its 5′ end, a sequence specific reverse primer and the universal fluorescently labeled M13 primer. For assaying microsatellite loci, PCR was carried out in a 10-μl reaction volume containing 15-ng template DNA, 0.5 unit Taq DNA polymerase (Ampli Taq Gold, Applied Biosystems, Foster City, CA, United States), 1X PCR buffer containing 1.5 mM MgCl_2_, 2 μM each of dNTPs, 0.4 μM of M13 tailed sequence specific forward primer, 1.2 μM of FAM labeled M13 primer, and 1.6 μM of the sequence specific reverse primer. Cycling was performed in a PCR machine (Mastercycler^®^ Gradient, Eppendorf, Germany) under the following conditions: initial denaturation at 94°C for 5 min followed by 30 cycles of 30 s at 94°C; 45 s at 56°C, 45 s at 72°C, followed by 8 cycles of 30 s at 94°C, 45 s at 53°C, 45 s at 72°C, and a final extension at 72°C for 10 min. Microsatellite alleles were detected following capillary electrophoresis of PCR products on an ABI Prism 3730 genetic analyzer (Applied Biosystems, Foster City, CA, United States). For capillary electrophoresis, the samples were prepared by mixing 1 μl of PCR product with 10 μl of Hi-Di formamide (Applied Biosystems, Foster City, CA, United States) and 0.1 μl of size standard [GENESCAN^®^ 400 HD (ROX) size standard, Applied Biosystems, Foster City, CA, United States]. The mixture was denatured at 94°C for 5 min and snap cooled on ice before subjecting to capillary electrophoresis. Allele sizing was performed by using the software GENESCAN 3.1.2 (Applied Biosystems, Foster City, CA, United States) using the “Local Southern Method” and default analysis settings by comparison with the internal size standard ROX 400. Allele calling and sorting was performed using the software GENEMAPPER V. 4 (Applied Biosystems, Foster City, CA, United States) and binning was performed manually.

The genetic diversity parameters, such as the number of alleles per locus, major allele frequency, gene diversity, heterozygosity, and polymorphism information content (PIC), were determined from the allelic data using the software PowerMarker Version 3.25 (Liu and Muse, 2005). We examined the population genetic structure of the rice samples using two methods: the genetic distance-based unweighted pair-group method with arithmetic average (UPGMA) clustering using PowerMarker (Liu and Muse, 2005) and model-based Bayesian clustering as implemented in the software STRUCTURE Version 2.3.4 (Pritchard et al., 2000). We performed the cluster analysis using the C. S. Chord genetic distance, and the resulting tree was viewed using the program TreeView Version 1.6.6. The tree was evaluated by bootstrapping on 1000 replications. The STRUCTURE analysis was run 10 times for each *K* (the number of clusters) from 1–20 using a model that allows for the admixture and correlated allele frequencies. The optimal *K*-value in the data set was determined according to the method of Evanno et al. (2005) as previously described (Varghese et al., 2014). STRUCTURE probabilistically generate ancestry coefficients totaled 1 for each genotype. Following the threshold used by Garris et al. (2005), we assigned a genotype with an ancestry coefficient > 0.8 into the corresponding population and treated a genotype as admixed if the coefficient was < 0.8. To determine the level of between-population molecular differentiation (pair-wise *F*_ST_), we performed an AMOVA using the software Arlequin Version 3.0 (Excoffier et al., 2005).

### Chloroplast Genotyping

We determined the chloroplast genotype by assaying the chloroplast locus ORF100 (Kanno et al., 1993) in 451 accessions, including 41 Njavara individuals, 12 Njavara-like individuals (**Supplementary Table 2**), 309 traditional cultivars (**Supplementary Table 3a**), 42 reference lines (**Supplementary Table 3c**), and 47 *O. rufipogon* accessions (**Supplementary Table 3d**). Following an initial standardization of different primer combinations, we finally chose a primer combination consisting of the forward primer (5′ CAACCCACCCCATAAAATTG 3′) reported by Garris et al. (2005) and the reverse primer (5′ CAGCCGAGGTCGTGGTAAATC 3′) used by Chuayjaeng and Volkaert (2006) for genotyping the ORF100 loci. PCR was carried out in a 10-μl reaction volume which contained 2X GoTaq colorless master mix (Promega, Madison, United States), 10 ng genomic DNA and 1 μM each of forward and reverse primers. DNA amplification was performed in Veritti^®^ 96-Well Thermal Cycler (Applied Biosystems, Foster City, CA, United States). Cycling conditions were 95°C for 2 min followed by 40 cycles of 30 s at 95°C, 40 s at 50°C, 40 s at 72°C, and concluding extension at 72°C for 5 min. The PCR assay produced a single amplicon at the ORF locus in the size range of 405–477 bp across different accessions used in the study. Initially, we sequenced the amplicon in a set of cultivars and confirmed the 69-bp deletion (deletion type chloroplast) in the smaller-sized amplicons and no such deletion (no-deletion type chloroplast) in the larger amplicons (Kanno et al., 1993; Garris et al., 2005; Chuayjaeng and Volkaert, 2006). Thereafter, we fractionated the PCR products on 1.5% agarose gels and directly scored the deletion or no-deletion alleles via a visual observation of ethidium bromide gels on a gel documentation system.

### Phylogenetic and Gene Genealogy Analysis

We randomly selected 19 unlinked expression sequence tag (EST)-based sequence tagged site (STS) loci (**Supplementary Table 4b**) from the pool of loci used previously for studying the phylogenetic relationship between rice and *O. rufipogon*. A single primer pair can retrieve sequence from a STS locus in both rice and *O. rufipogon*. These STS loci follow neutral expectations and are appropriate for a phylogenetic study (Caicedo et al., 2007; Huang P.U. et al., 2012). We sequenced the chosen loci in 186 samples, including 43 Njavara individuals, 12 Njavara-like individuals (**Supplementary Table 2**), 69 traditional cultivars (**Supplementary Table 3a**), 15 reference lines (**Supplementary Table 3c**), and 47 *O. rufipogon* accessions (**Supplementary Table 3d**). We custom-synthesized oligonucleotide primers for PCR amplification of the STS loci with Sigma (Sigma Genosys, Bangaluru, India) (**Supplementary Table 4b**). PCR was carried out in a 10-μl reaction volume which contained 1X PCR buffer (150 mM Tris–HCl, pH-8; 500 mM KCl), 0.2 mM each dNTPs (dATP, dGTP, dCTP, and dTTP), 2.5 mM MgCl_2_, 20 ng genomic DNA, 1 unit of AmpliTaq Gold DNA polymerase enzyme (Applied Biosystems, Foster City, CA, United States), 0.1 mg/ml BSA and 4% DMSO, and 5 pM each of forward and reverse primers. DNA amplification was performed in a Mastercycler^®^ Gradient Thermal Cycler (Eppendorf, Germany). Cycling conditions were 94°C for 5 min followed by 40 cycles of 94°C (30 s), 55°C (30 s–1 min), 72°C (30 s–1 min), and concluding extension at 72°C (5 min). The PCR products were cleaned using ExoSAP-IT^TM^ (USB Corporation, United States) and subjected to bidirectional sequencing using a BigDye^®^ Terminator v3.1 Cycle sequencing kit (Applied Biosystems, Foster City, CA, United States), which used the primers employed for amplification. The cleaned sequenced PCR products were run on an ABI Prism 3730 Genetic Analyzer (Applied Biosystems, Foster City, CA, United States). The sequence quality was assessed using the software Sequence Scanner v1 (Applied Biosystems, Foster City, CA, United States). Sequence editing and alignment were conducted using the software Geneious R.6 (Kearse et al., 2012). We subjected the sequences to homology searches at the National Center for Biotechnology Information (NCBI) database using the Basic Local Alignment Search Tool (BLAST) algorithm to confirm the authenticity of the obtained sequence.

We estimated the level of nucleotide diversity (Watterson’s 𝜃 and π) and the number of haplotypes (h) and haplotype diversity (Hd) for each locus across the 186 samples using the software DnaSP Version 5.1 (Librado and Rozas, 2009) which was also used to perform the test of deviation from neutral expectations separately for rice and *O. rufipogon* samples based on Tajima’s D, Fu and Li’s D, and Fu and Li’s F^∗^ statistics. We reconstructed the phylogenetic relationships between the 186 samples based on the concatenated sequence of the 19 STS loci using the software PAUP^∗^ Version 4.0 (Swofford, 2002) following neighbor-joining method. Bootstrapping was performed (500 replicates) to assess the robustness of the clustering in the trees.

The genealogical network of haplotypes from genes can better resolve reticulations, such as hybridizations and introgressions, observed in the evolutionary trajectories of a taxon and interconnect the descendant genes with the persistent ancestors (Posada and Crandall, 2001). We constructed a statistical parsimony network for the STS haplotypes using the software TCS Version 1.21 (Clement et al., 2000). The statistical parsimony algorithm estimates the number of differences in single nucleotide substitutions between haplotypes and then generates a pair-wise distance matrix between haplotypes based on the probability of parsimony for pair-wise differences until the probability exceeds 0.95. The phylogenetic relationship between the haplotypes is depicted in the form of an acyclic graph (network).

NCBI accession numbers of DNA sequences generated in this study are provided in the **Supplementary Tables**.

## Results and Discussion

### Njavara Is Distinct From the Canonical Sub-Groups Known in Rice

First, we examined the genetic interrelationship between Njavara and other rice cultivars. We analyzed 589 rice individuals, including 213 genetically distinct individuals chosen from 100 Njavara collections (**Figure 1A** and **Supplementary Tables 2, 3a–c**). In addition to the canonical ancestral sub-groups (Garris et al., 2005), a UPGMA cluster analysis of 589 rice individuals (**Figure 1B** and **Supplementary Figure 1**) using the allelic data generated by 70 microsatellite loci (**Supplementary Tables 4a, 5a**) produced a non-canonical group confined within 170 Njavara individuals belonging to 78 collections (**Figure 1B**, **Supplementary Figure 1** and **Supplementary Table 2**). The pattern of population sub-divisions produced by the Bayesian algorithm STRUCTURE in a set of increasing *K*-value is given in **Figure 1C**. Initially, all the Njavara individuals together produced a single cluster at *K* = 2. Three clusters were discernible within Njavara germplasm at *K* = 8. Overall population sub-divisions produced by the STRUCTURE were corresponded well with the clustering patters in UPGMA dendrogram at *K* = 15 (**Figures 1B,C**, **Supplementary Figure 1** and **Supplementary Table 2**). Of the six genotypic classes identified earlier in the Njavara germplasm (Sreejayan et al., 2011; Varghese et al., 2014), five classes, namely, Short black I (SB I), Short black III (SB III), and Intermediate yellow (IY), produced distinct groups, and the Short yellow (SY), and Long yellow (LY) genotypes produced distinct sub-groups under one major node (hereafter referred to as the LY/SY group) in the UPGMA dendrogram and the STRUCTURE bar plot (**Figures 1B,C**, **Supplementary Figure 1** and **Supplementary Table 2**). The pair-wise *F*st-values between Njavara and the canonical sub-groups differed significantly (*p* < 0.001) (**Supplementary Table 5b**). In addition, the STRUCTURE analysis classified Njavara individuals into respective genotypic classes with a high probability (< 90%) (**Supplementary Table 6**), suggesting the genetic purity of Njavara individuals. Together, these results illustrate that the Njavara represents a clade distinct from the known ancestral sub-groups (Garris et al., 2005) in the rice gene pool.

A total of 43 individuals from the Njavara collections showed high genetic affinity to different canonical ancestral sub-groups (**Supplementary Figure 1** and **Supplementary Tables 2, 6**) and will hereafter be referred to as “Njavara like” individuals. Short black II, the sixth genotypic class identified in the previous study (Sreejayan et al., 2011), was identified as *aus* in the present study (**Supplementary Figure 1** and **Supplementary Table 6**). Hence, we included this class also under Njavara-like individuals.

### Njavara Is Likely an Extant Early Domesticate

**FIGURE 2:**
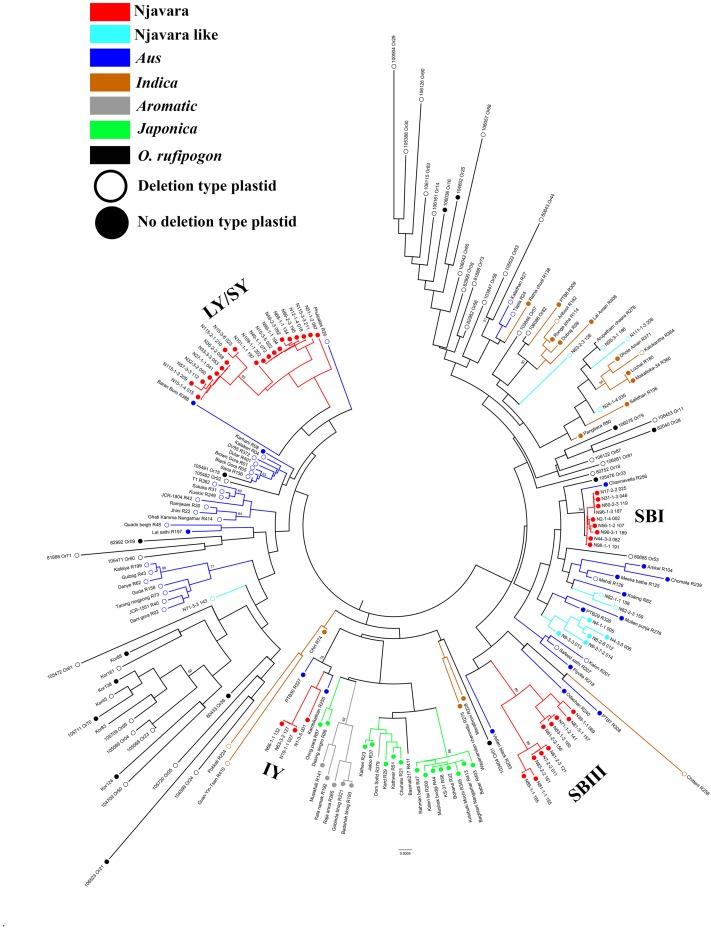
Njavara individuals separate into distinct sub-clades in the evolutionary tree of rice. The figure shows the neighbor-joining evolutionary tree of 139 rice individuals and 47 accessions of the progenitor *O. rufipogon* based on the combined sequences of 19 STS loci. The Njavara genotypic classes Intermediate yellow (IY), Long yellow/Short yellow (LY/SY), Short black I (SB I) and Short black III (SB III) produced distinct sub-clades and are labeled in the tree. The ancestral sub-groups *aus, indica, aromatic*, and *japonica*, and the Njavara like and *O. rufipogon* were also formed distinct clades and are color coded. The no-deletion chloroplast is indicated by a closed circle at the end of the node, and the deletion type chloroplast by an open circle. Bootstrap values > 50% is given at the respective nodes. Sample name is given at the end of each node. The individual name and number, as in **Supplementary Table 2**, is used to name the Njavara individuals. The cultivar name and IRGC number followed by the RGCB collection serial number, as in **Supplementary Table 3**, is used for naming traditional cultivar and *O. rufipogon*, respectively. R or Or before the RGCB collection serial number indicates rice or *O. rufipogon*, respectively.

Next, we examined the phylogeographic evolutionary history of Njavara following a multiple gene genealogical approach. For this purpose, we retrieved sequences of a set of 19 unlinked EST-based STS loci (**Supplementary Table 4b**) from 186 samples, including 139 rice individuals and 47 accessions of *O*. *rufipogon* (**Supplementary Tables 2, 3a,c,d**). The test of neutrality using different parameters (Tajima’s D, Fu and Li’s D, and Fu and Li’s F^∗^ statistics) showed that the STS loci used in the study follow neutral expectations (**Supplementary Table 7**). We combined the sequence of 19 STS loci (**Supplementary Table 7**) and generated a neighbor-joining phylogenetic tree (**Figure 2**). In the phylogenetic tree, the individuals belonging to the Njavara genotypic classes SB I, SB III, and LY/SY were separated into distinct sub-clades and entered into three separate major clades consisting of *aus* cultivars, whereas the individuals belonging to the IY genotype produced another distinct sub-clade and entered into another major clade consisting of *japonica* and *aromatic* cultivars (**Figure 2**).

**FIGURE 3:**
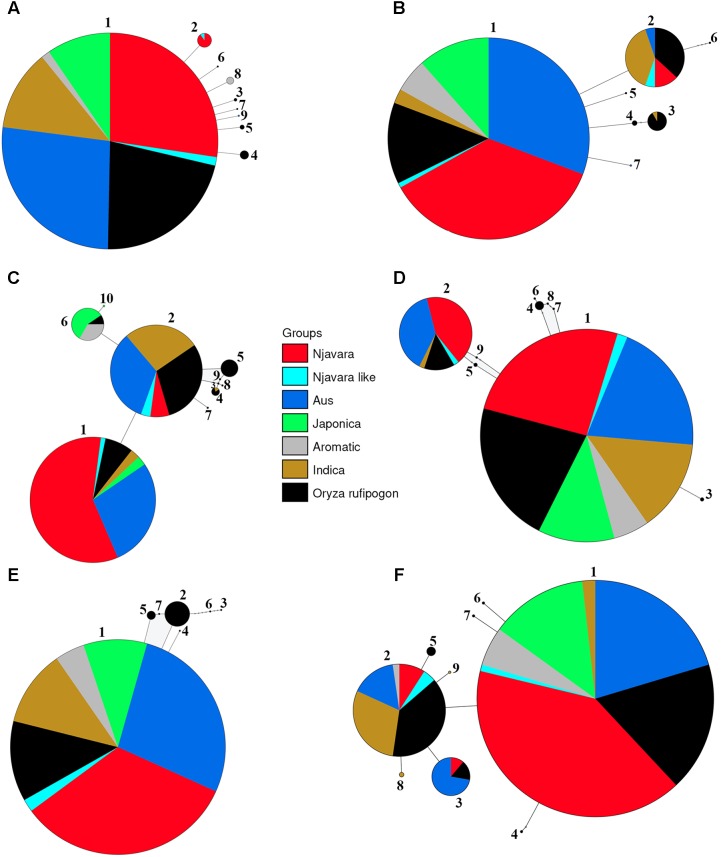
Representative statistical parsimony haplotype network of 139 rice samples and 47 *O. rufipogon* accessions for the STS loci STS 011 **(A)**, STS 025 **(B)**, STS 047 **(C)**, STS 070 **(D)**, STS 108 **(E)**, and STS 080 **(F)**. The size of each circle is proportional to the haplotype frequency. The area of color coded segment in the circles is proportional to the frequency of different populations sharing a haplotype and the colors are deciphered in the key. Small open circles represent missing haplotypes. Haploid number according to **Supplementary Table 8** is given for each circle.

Genealogical analyses were conducted for each STS locus separately. The number of haplotypes detected at a locus in the 186 samples varied between five in STS 087 and 31 in STS 085 (**Supplementary Table 7**). For most loci, the genealogical analysis produced a central haplotype shared among most of the cultivars belonging to the ancestral sub-groups, Njavara accessions and a set of *O*. *rufipogon* populations originating mostly from Central India to Southeast Asian countries and a few peripheral ones separated by few mutational steps (**Figure 3** and **Supplementary Tables 8, 9**). The high frequency central haplotypes are most likely ancestral ones and are probably fixed in the rice during the domestication processes. Genetic analysis using domestication related genes shows introgression of domestication alleles from fully domesticated *japonica* into other ancestral groups during their domestication processes (Molina et al., 2011; He et al., 2011; Huang X. et al., 2012; Yang et al., 2012). The possible introgression of alleles from *japonica* into rice accessions used in the analysis could not be determined as we did not include the domestication alleles in the analysis. A close genetic relationship between the haplotypes phased from *O. rufipogon* and the other rice cultivars were evident in the network (**Figure 3**). Gene flow from self-pollinating rice to out crossing *O. rufipogon* is wide spread in natural habitats (Song et al., 2006; Kim et al., 2016) and this provides reticulate relationships between *O. rufipogon* and cultivated rice in phylogenetic and genealogical analysis (Londo et al., 2006; Huang X. et al., 2012).

Thus, the genetic and gene genealogical treatments performed in the present study together with the results of the morphological analysis reported previously (Varghese et al., 2014) reveal two characteristic features of Njavara. First, a cohesiveness is observed between the four genotypic classes of Njavara (**Figure 1B** and **Supplementary Figure 1**) at the genetic and morphological levels despite their distinct positions in the phylogenetic tree (**Figure 2**). Second, Njavara retains a distinct and smaller morphological architecture (Varghese et al., 2014). Both of these genetic characteristics must represent a corollary of an on-farm stabilizing selection traditionally performed by farmers on Njavara using 60-days maturity as the selection criterion (Sreejayan et al., 2011; Varghese et al., 2014). Because, the stabilizing selection leads to morphological convergence by preventing the selected trait from differentiating across environments (Latta, 1998) and deepens the genetic differentiation between the selected and non-selected populations by enhancing the divergence in loci experiencing balancing selection and linked neutral loci (Charlesworth et al., 1997). The previous study has reported significantly smaller (*p* < 0.05) characteristics with respect to the yield-related traits, including grain dimensions in Njavara than the syntopic traditional cultivars (Varghese et al., 2014). The heading date and several yield-related traits are co-regulated by pleiotropic quantitative trait loci (QTL) in rice (Xue et al., 2008; Wei et al., 2010; Zhang et al., 2010; Yan et al., 2011; Cai et al., 2012; Varghese et al., 2014). Therefore, in addition to the heading date, the stabilizing selection performed by farmers of Njavara using 60-days maturity has concomitantly preserved the allele complements that govern the morphological architecture also since ancient times, resulting in the retention of the small plant architecture that is presumably a characteristics of early domesticates (Fuller et al., 2010; Castillo et al., 2016). Thus, Njavara provides an example of how an “unconscious selection” (Zohary, 2004) performed by farmers preserved the genetic purity and morphological characteristics of a cultivar over millennia. However, on what basis people in the ancient days have specifically chosen short duration cultivars emanating from different phylogenetically distinct groups for medicinal applications is not understood from the present data. A comparative global metabolome profiling between Njavara and other cultivars may provide evidence to such queries.

Intriguingly, the Njavara individuals invariably had a no-deletion chloroplast genotype (**Figure 2** and **Supplementary Table 2**). Most of the *aus* cultivars entered into the major clade that consisted of the SB I and SB III genotypes were also of the no-deletion type (**Figure 2** and **Supplementary Tables 3a,c**). The Njavara genotypes produced many haplotypes shared only with *aus* (**Supplementary Tables 8, 9**). Recent research has increasingly shown that *aus* is genetically complex (Travis et al., 2015) and its origin is distinct (Kim et al., 2016; Wang et al., 2016). Results of the present study together with the results of Civáň et al. (2016), who found that *aromatic* may be a product of hybridization between *japonica* and *aus*, prompt us to infer that Njavara is closest to *aus* and the no-deletion cultivars related to *aus*, such as Njavara, constitute one of the earliest domesticates of rice in India.

### Geographic Affinity of Njavara Lies With Central India-Southeast Asia Region

The genealogical analysis suggests that the geographic center of origin of Njavara encompasses Central India to Southeast Asia because its genetic affinity mostly lies with the *O. rufipogon* populations from this region (**Supplementary Tables 8, 9**). This finding raises a pertinent question: how did a rice strain domesticated in Central India-Southeast Asian region reach Kerala and become preserved in this state? In ancient India, the Gangetic plains (**Figure 1**), which encompassed Magadha (modern Bihar state in India), the seat of the Iron Age Maurya dynasty (ca. 323–185 B.C.), were an epicenter of rice cultivation (Fuller et al., 2010) and Buddhist doctrines (Zysk, 1998). The development and spread of Buddhism, Ayurveda, and rice cultivation are entwined (Zysk, 1998; Shaw et al., 2007; Ninivaggi, 2010). Mauryan Emperor Asoka (ca. 269–23 B.C.), who spearheaded the spread of Buddhism and Ayurveda (Zysk, 1998; Ninivaggi, 2010) proclaimed that Buddhist ascetics required the importation and planting of medicinal herbs wherever these herbs were not found (Zysk, 1998). Rice was introduced to South India during the spread of Buddhism (Shaw et al., 2007). Buddhism flourished in Kerala between the third century B.C. and the eighth century A.D. (Menon, 2007). Ayurveda also flourished in these periods (Menon, 2007) because Ayurveda medicine and healing were integral parts of Buddhist monasticism (Zysk, 1998). The people of Kerala have been ritualistically preserving Njavara (Sreejayan et al., 2011) which may have reached this state concomitant with Ayurveda. Njavara grain-based treatments, such as *Shashtikapindasweda* (Sanskrit) or *Njavarakizhi* (Malayalam) and *Shashtikaannalepa* (Sanskrit) or *Njavaratheppu* (Malayalam), that belong to a group of treatments called *Keraliya Panca Karma* (Kerala specialty of *Panca Karma* treatments) and developed historically in Kerala by *Ashtavaidyas* are continued to be the acclaimed therapies for various illnesses even today (Singh, 1992; Sreejayan et al., 2011).

## Conclusion

Our study circumscribed Njavara gene pool in rice genetic resources, and demonstrates the value of linguistic-genetic approaches in the domestication research of rice. Njavara is distinct from the known canonical ancestral subgroups in rice (Garris et al., 2005) and is originated in the central India-Southeast Asia region. It is likely a genetically pure extant early domesticate still being cultivated in India. Also, Njavara provide a rare example of traditional prudence in on-farm selection that preserved a cultivar in pure form over millennia because of its applications in healthcare. Njavara may constitute a good organism to investigate the genomic changes associated with domestication and on-farm evolutionary trajectories in rice. The circumscription of the Njavara gene pool achieved in this study may give an impetus to the discovery of genes for the biofortification of rice because, consistent with the traditional claims (Bhishagratna, 1907; Van Loon, 2003), empirical research has recently reported medicinally active and nutritionally rich molecules in Njavara grains (Deepa et al., 2008; Jung et al., 2014).

## Author Contributions

GT designed and supervised this study. MJ, RDR, and RM performed the microsatellite and STS genotyping. MRV and JB conducted the statistical analysis. GV, RY, ONS, BCP, JCR, and SLK collected the rice germplasm.

## Conflict of Interest Statement

The authors declare that the research was conducted in the absence of any commercial or financial relationships that could be construed as a potential conflict of interest.

## Footnotes

1 http://www.gramene.org

## References

[B1] Bhishagratna K. L. (ed.) (1907). “Sutrasthanam,” in *An English Translation of the Sushruta Samhita, Based on Original Sanskrit Text* Vol. 1 (London: Forgotten Books).

[B2] Burrow T. Emeneau M. B. (1961). *A Dravidian Etymological Dictionary.* Oxford: Oxford University Press.

[B3] Cai H. Y. Diao S. He Y. G. Zhang L. P. Liu S. J. Zhu Y. G. (2012). Genetic and physical mapping of qHY-8, a pleiotropic QTL for heading date and yield-related traits in rice. *Euphytica* 184 109–118. 10.1007/s10681-011-0581-0

[B4] Caicedo A. L. Williamson S. H. Hernandez R. D. Boyko A. Fledel-Alon A. Yort T. L. (2007). Genome-wide patterns of nucleotide polymorphism in domesticated rice. *PLoS Genet.* 3:e163. 10.1371/journal.pgen.0030163 17907810 PMC1994709

[B5] Castillo C. C. Tanaka K. Sato Y. I. Ishikawa R. Bellina B. Higham C. (2016). Archaeogenetic study of prehistoric rice remains from Thailand and India: evidence of early japonica in South and Southeast Asia. *Archaeol. Anthropol. Sci.* 8 523–543. 10.1007/s12520-015-0236-5

[B6] Charlesworth B. Nordborg M. Charlesworth D. (1997). The effects of local selection, balanced polymorphism and background selection on equilibrium patterns of genetic diversity in subdivided populations. *Genet. Res.* 70 155–174. 10.1017/S0016672397002954 9449192

[B7] Chuayjaeng S. Volkaert H. (2006). Chloroplast diversity and phylogeny in wild and cultivate rice (*Oryza* spp.). *Kasetsart J.* 40 306–313.

[B8] Civáň P. Craig H. Cymon J. Cox C. J. Brown T. A. (2016). Three geographically separate domestications of Asian rice. *Nat. Plants* 1:15164. 10.1038/nplants.2015.164 27251535 PMC4900444

[B9] Clement M. Posada D. Crandall K. A. (2000). TCS: a computer program to estimate gene genealogies. *Mol. Ecol.* 9 1657–1660. 10.1046/j.1365-294x.2000.01020.x 11050560

[B10] Deepa G. Singh V. Naidu K. A. (2008). Nutrient composition and physicochemical properties of Indian medicinal rice- Njavara. *Food Chem.* 106 165–171. 10.1016/j.foodchem.2007.05.062

[B11] Evanno G. Regnaut S. Goudet J. (2005). Detecting the number of clusters of individuals using the software STRUCTURE: a simulation study. *Mol. Ecol.* 14 2611–2620. 10.1111/j.1365-294X.2005.02553.x 15969739

[B12] Excoffier L. Laval G. Schneider S. (2005). Arlequin (version 3.0): an integrated software package for population genetics data analysis. *Evol. Bioinform.* 1 47–50. 10.1177/117693430500100003 19325852 PMC2658868

[B13] Fuller D. Q. Sato Y. I. Castillo C. Qin L. Weisskopf A. R. Kingwell-Banham E. J. (2010). Consilience of genetics and archaeobotany in the entangled history of rice. *Archaeol. Anthropol. Sci.* 2 115–131. 10.1007/s12520-010-0035-y

[B14] Garris A. J. Tai T. H. Coburn J. Kresovich S. McCouch S. (2005). Genetic structure and diversity in *Oryza sativa* L. *Genetics* 169 1631–1638. 10.1534/genetics.104.035642 15654106 PMC1449546

[B15] Glaszmann J. C. (1987). Isozymes and classification of Asian rice varieties. *Theor. Appl. Genet.* 74 21–30. 10.1007/BF00290078 24241451

[B16] Gross B. L. Zhao Z. (2014). Archaeological and genetic insights into the origins of domesticated rice. *Proc. Natl. Acad. Sci. U.S.A.* 111 6190–6197. 10.1073/pnas.1308942110 24753573 PMC4035933

[B17] He Z. Zhai W. Wen H. Tang T. Wang Y. Lu X. (2011). Two evolutionary histories in the genome of rice: the role of domestication genes. *PLoS Genet.* 7:e1002100. 10.1371/journal.pgen.1002100 21695282 PMC3111475

[B18] Huang P. U. Molina J. Flowers J. M. Rubinstein S. Jackson S. A. Purugganan M. D. (2012). Phylogeography of Asian wild rice, *Oryza rufipogon*: a genome-wide view. *Mol. Ecol.* 21 4593–4604. 10.1111/j.1365-294X.2012.05625.x 22646149

[B19] Huang X. Kurata N. Wang Z. X. Wang A. Zhao Q. Zhao Y. A. (2012). A map of rice genome variation reveals the origin of cultivated rice. *Nature* 490 497–501. 10.1038/nature11532 23034647 PMC7518720

[B20] Jung Y. S. Kim D. H. Hwang J. Y. Yun N. Y. Lee Y. H. Han S. B. (2014). Anti-inflammatory effect of tricin 4′-O-(threo-β-guaiacylglyceryl) ether, a novel flavonolignan compound isolated from Njavara on in RAW264.7 cells and in ear mice edema. *Toxicol. Appl. Pharmcol.* 277 67–76. 10.1016/j.taap.2014.03.001 24631338

[B21] Kanno A. Watanabe N. Nakamura I. Hirari A. (1993). Variations in chloroplast DNA from rice (*Oryza sativa*): differences between deletions mediated by short direct-repeat sequences within a single species. *Theor. Appl. Genet.* 86 579–584. 10.1007/BF00838712 24193706

[B22] Kearse M. Moir R. Wilson A. Stones-Havas S. Cheung M. Sturrock S. (2012). Geneious basic: an integrated and extendable desktop software platform for the organization and analysis of sequence data. *Bioinformatics* 28 1647–1649. 10.1093/bioinformatics/bts199 22543367 PMC3371832

[B23] Kim H. J. Jung J. Singh N. Greenberg A. Doyle J. J. Tyagi W. (2016). Population dynamics among six major groups of the *Oryza rufipogon* species complex, wild relative of cultivated Asian rice. *Rice* 9:56. 10.1186/s12284-016-0119-0 27730519 PMC5059230

[B24] Latta R. G. (1998). Differentiation of allelic frequencies at quantitative trait loci affecting locally adaptive traits. *Am. Nat.* 151 283–292. 10.1086/286119 18811359

[B25] Librado P. Rozas J. (2009). DnaSP v5: a software for comprehensive analysis of DNA polymorphism data. *Bioinformatics* 25 1451–1452. 10.1093/bioinformatics/btp187 19346325

[B26] Liu K. Muse S. V. (2005). PowerMarker: an integrated analysis environment for genetic marker analysis. *Bioinformatics* 21 2128–2129. 10.1093/bioinformatics/bti282 15705655

[B27] Londo J. P. Chiang Y. C. Hung K. H. Chiang T. Y. Schaal B. A. (2006). Phylogeography of Asian wild rice, *Oryza rufipogon*, reveals multiple independent domestications of cultivated rice, *Oryza sativa*. *Proc. Natl. Acad. Sci. U.S.A.* 103 9578–9583. 10.1073/pnas.0603152103 16766658 PMC1480449

[B28] Menon A. S. (ed.) (2007). *A Survey of Kerala History.* Kottayam: DC Books.

[B29] Molina J. Sikora M. Garud N. Flowers J. M. Rubinstein S. Reynolds A. (2011). Molecular evidence for a single evolutionary origin of domesticated rice. *Proc. Natl. Acad. Sci. U.S.A.* 108 8351–8356. 10.1073/pnas.1104686108 21536870 PMC3101000

[B30] Nene Y. L. (2005). Rice research in South Asia through Ages. *Asian Agri-Hist.* 9 85–106.

[B31] Ninivaggi F. J. (ed.) (2010). *Ayurveda: A Comprehensive Guide to Traditional Indian Medicine for the West*. Plymouth: Rowman and Littlefield Publishers, Inc.

[B32] Posada D. Crandall K. A. (2001). Intraspecific gene genealogies: trees grafting into networks. *Trends Ecol. Evol.* 16 37–45. 10.1016/S0169-5347(00)02026-7 11146143

[B33] Pritchard J. K. Stephens M. Donnelly P. (2000). Inference of population structure using multilocus genotype data. *Genetics* 155 945–959. 10835412 10.1093/genetics/155.2.945 PMC1461096

[B34] Roy S. Marndi B. C. Mawkhlieng B. Banerjee A. Yadav R. M. Misra A. K. (2016). Genetic diversity and structure in hill rice (*Oryza sativa* L.) landraces from the North-Eastern Himalayas of India. *BMC Genet.* 17:107. 10.1186/s12863-016-0414-1 27412613 PMC4944464

[B35] Saxena A. Prasad V. Singh I. B. Chauhan M. S. Hasan R. (2006). On the Holocene record of phytoliths of wild and cultivated rice from Ganga Plain: evidence for rice-based agriculture. *Curr. Sci.* 90 1547–1552.

[B36] Schuelke M. (2000). An economic method for the fluorescent labeling of PCR fragments. *Nat. Biotechnol.* 18 233–234. 10.1038/72708 10657137

[B37] Shaw J. Sutcliffe J. V. Lloyd-Smith L. Schwenninger J. L. Chauhan M. S. Misra O. P. (2007). Ancient irrigation and Buddhist history in Central India: optically stimulated luminescence dates and pollen sequences from the Sanchi Dams. *Asian Perspect.* 46 166–201. 10.1353/asi.2007.0011

[B38] Singh R. H. (ed.) (1992). *Panca Karma Therapy: Ancient Classical Concepts, Traditional Practices, and Recent Advances*. Varanasi: Chowkhamba Sanskrit Series Office.

[B39] Song Z. Zhu W. Rong J. Xu X. Chen J. Lu B. R. (2006). Evidences of introgression from cultivated rice to *Oryza rufipogon* (Poaceae) populations based on SSR fingerprinting: implications for wild rice differentiation and conservation. *Evol. Ecol.* 20 501–522. 10.1007/s10682-006-9113-0

[B40] Sreejayan U. Suresh Kumar V. Varghese G. Jacob T. M. Thomas G. (2011). Stratification and population structure of the genetic resources of ancient medicinal rice (*Oryza sativa* L.) landrace Njavara. *Genet. Resour. Crop Evol.* 58 697–711. 10.1007/s10722-010-9613-1

[B41] Swofford D. L. (2002). *PAUP^∗^. Phylogenetic Analysis using Parsimony (and other methods) Version 4*. Sunderland, MA: Sinauer Associates.

[B42] Travis A. J. Norton G. J. Datta S. Sarma R. Dasgupta T. Savio F. L. (2015). Assessing the genetic diversity of rice originating from Bangladesh, Assam and West Bengal. *Rice* 8:35. 10.1186/s12284-015-0068-z 26626493 PMC4667538

[B43] Van Loon G. (ed.) (2003). *Charaka Samhita: Handbook on Ayurveda*, Vol. I. Morrisville, NC: Lulu Inc.

[B44] Varghese G. Jose M. Dinesh Raj R. Bocianowski J. Thomas G. Omanakumari N. (2014). Quantitative and molecular analyses reveal a deep genetic divergence between the ancient medicinal rice (*Oryza sativa*) Njavara and syntopic traditional cultivars. *Ann. Appl. Biol.* 164 95–106. 10.1111/aab.12083

[B45] Wang H. Xu X. Vieira F. G. Xiao Y. Li Z. Wang J. (2016). The power of inbreeding: NGS-based GWAS of rice reveals convergent evolution during rice domestication. *Mol. Plant* 9 975–985. 10.1016/j.molp.2016.04.018 27179918

[B46] Wang W. Mauleon R. Hu Z. Chebotarov D. Tai S. Wu Z. (2018). Genomic variation in 3,010 diverse accessions of Asian cultivated rice. *Nature* 557 43–49. 10.1038/s41586-018-0063-9 29695866 PMC6784863

[B47] Wei X. Xu J. Guo H. Jiang L. Chen S. Yu C. (2010). DTH8 suppresses flowering in rice, influencing plant height and yield potential simultaneously. *Plant Physiol.* 153 1747–1758. 10.1104/pp.110.156943 20566706 PMC2923886

[B48] Xue W. Xing Y. Weng X. Zhao Y. Tang W. Wang L. (2008). Natural variation in Ghd7is an important regulator of heading date and yield potential in rice. *Nat. Genet.* 40 761–767. 10.1038/ng.143 18454147

[B49] Yan W. H. Wang P. Chen H. X. Zhou H. J. Li Q. P. Wang C. R. (2011). A major QTL, Ghd8, plays pleiotropic roles in regulating grain productivity, plant height, and heading date in rice. *Mol. Plant* 4 319–330. 10.1093/mp/ssq070 21148627

[B50] Yang C. C. Kawahara Y. Mizuno H. Wu J. Matsumoto T. Itoh T. (2012). Independent domestication of Asian rice followed by gene flow from japonica to indica. *Mol. Biol. Evol.* 29 1471–1479. 10.1093/molbev/msr315 22319137

[B51] Zhang Y. S. Wang J. B. Xu C. G. Xing Y. Z. (2010). Molecular dissection of genetic basis of significant correlation among five morphological traits in rice (*Oryza sativa* L.). *Chin. Sci. Bull.* 55 3154–3160. 10.1007/s11434-010-1022-5

[B52] Zohary D. (2004). Unconscious selection and the evolution of domesticated plants. *Econ. Bot.* 58 5–10. 10.1663/0013-00012004058

[B53] Zysk K. G. (ed.) (1998). *Asceticism and Healing in Ancient India: Medicine in the Buddhist Monastery*. New Delhi: Motilal Banarsidass.

