# Supplementary figures and images for: The Prehistoric Indian Ayurvedic Rice Shashtika Is an Extant Early Domesticate With a Distinct Selection History
Source: Front Plant Sci. 2018 Aug 14;9:1203. doi: 10.3389/fpls.2018.01203 (PMC6102419; doi:10.3389/fpls.2018.01203)

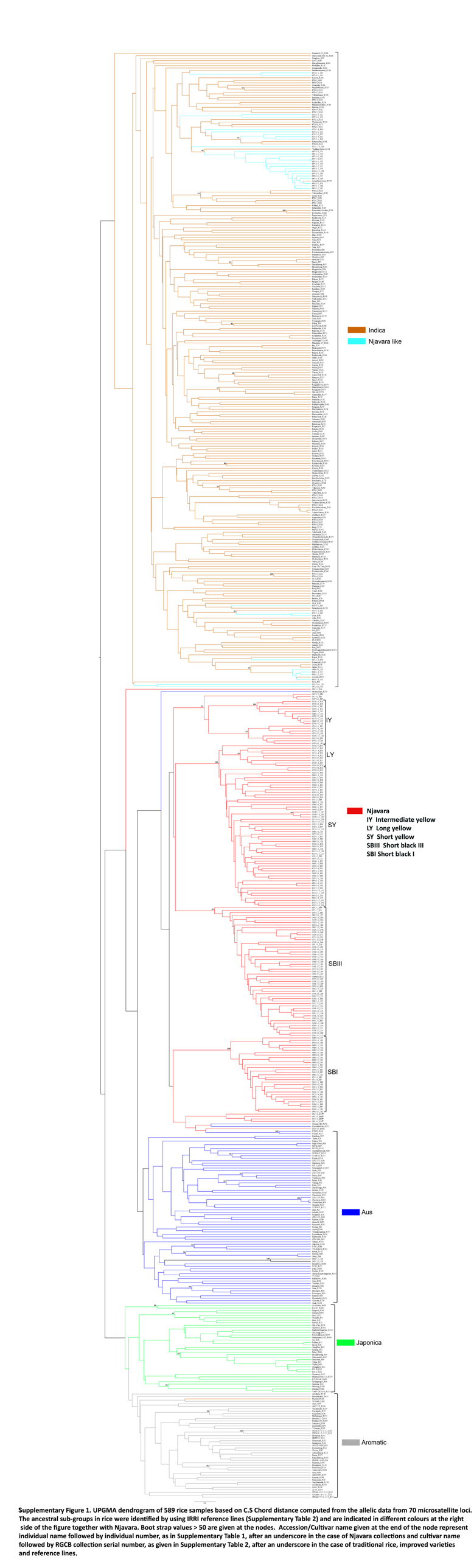

Supplement: Supplementary file 1 [file Image_1.JPEG]
